# Innovative tools for assessing risks for severe adverse events in areas of overlapping *Loa loa* and other filarial distributions: the application of micro-stratification mapping

**DOI:** 10.1186/1756-3305-7-307

**Published:** 2014-07-03

**Authors:** Louise A Kelly-Hope, Jorge Cano, Michelle C Stanton, Moses J Bockarie, David H Molyneux

**Affiliations:** 1 Centre for Neglected Tropical Diseases, Department of Parasitology, Liverpool School of Tropical Medicine, Liverpool, UK; 2 Faculty of Infectious and Tropical Diseases, London School of Hygiene & Tropical Medicine, London, UK

**Keywords:** Loa loa, RAPLOA, Onchocerciasis, Lymphatic filariasis, Democratic Republic of Congo, Severe adverse events, Micro-stratification mapping

## Abstract

**Background:**

The wide distribution of *Loa loa* infection (loiasis) throughout the Democratic Republic of Congo (DRC) is a major obstacle to the plans to eliminate onchocerciasis and lymphatic filariasis (LF) because the standard drug regime is dependent on ivermectin, which cannot be used in co-endemic areas due to the risk of severe adverse events (SAEs). A better understanding of the micro-epidemiology, overlapping low and high risk areas, and how they relate to SAEs is critical to ensure safe and effective treatment.

**Findings:**

Based on published data from the Bas Congo Province in DRC, this study used geographical information systems (GIS) to re-map and analyse onchocerciasis and loiasis prevalence (<20%, 20 to 40%, >40%) at 144 sites in relation to health district areas reporting SAEs. The new maps highlighted the contrasting patterns of the high prevalence sites, and significant geographical overlap between low onchocerciasis and high loiasis sites. Statistical analyses found that sites with medium to high loiasis prevalence were 10 to 16 times more likely to be in a SAE area than those with low prevalence of loiasis. Sites where both onchocerciasis and loiasis prevalence was >20% were also associated with SAE areas.

**Conclusions:**

Collaborative efforts between the national onchocerciasis and LF programmes are critical as plans to scale interventions are moving forward and thus, alternative strategies needed in loiasis co-endemic areas which may include the new *L. loa* test and treat strategy using the Cellscope, or interventions such as integrated vector management, or anti *Wolbachia* therapy using doxycycline.

## Findings

### Background

The endemicity and wide distribution of *Loa loa* filariasis (loiasis) in Central Africa is a major concern for national onchocerciasis programmes supported by the African Programme for Onchocerciasis Control (APOC) and non-governmental development organisation (NGDOs) due to the increased risk of severe adverse events (SAEs) associated with the mass distribution of ivermectin (Mectizan®) [1,2]. *L. loa* is transmitted to humans by Tabanid flies of the genus *Chrysops* spp. which are predominately confined to the tropical forests of Africa. Although most *L. loa* infected individuals are asymptomatic, infection can cause tropical eye worm, or Calabar swelling; progressive neurologic decline, encephalopathy and death have been reported in individuals after taking ivermectin for onchocerciasis. Individuals with high *L. loa* microfilarial (Mf) densities of ≥ 30,000 (Mf/ml blood) are considered to be most at risk of SAEs [3].

APOC has targeted the control of onchocerciasis (river blindness) in Africa since 1995 using ivermectin, which is an effective microfilaricide (donated by Merck & Co.Inc) [4]. The disease is caused by the *Onchocerca volvulus* parasite and transmitted to humans by river breeding *Simulium* blackflies [5]. Infection can result in visual impairment or blindness and serious skin disease including sub-cutaneous nodules where adult worms reside. Over the past decade the community-directed treatment with ivermectin (CDTi) strategy has been used to target meso-hyperendemic areas, defined by community nodule prevalence ≥20% using the rapid epidemiological mapping of onchocerciasis (REMO) survey tool [6]. The CDTi priority maps have been used extensively by national programmes to define project areas, however, now as APOC moves its focus from the ‘*control’* to the ‘*elimination’* of onchocerciasis [7,8] significant challenges arise, particularly in areas hypo-endemic <20% for onchocerciasis where *L. loa* transmission occurs [9].

Defining the extent of loiasis co-endemicity on a large scale has been possible by the development of the rapid assessment procedure for loiasis (RAPLOA) [10,11], which was able to correlate the prevalence of eye worm history (most recent episode lasting < 7 days) with the *L. loa* microfilaraemia [12]. Communities with ≥40% eye worm history prevalence were considered to be most at risk of SAEs as this was found to correspond approximately to 20% Mf prevalence, with 5% of individuals estimated to have ≥ 8000 mf/ml, and 2% of individuals ≥ 30,000 mf/ml in their blood [10-13]. However, *L. loa* prevalence models also indicate that some communities where >20% of individuals have a history of eye worm may also be at risk of *L. loa* encephalopathy as they harbour similar high *L. loa* Mf densities [9]. This has important implications for defining the critical threshold for safe and effective treatment, and if set at a RAPLOA prevalence of 20% would substantially reduce the risk of SAEs. However, this would also mean that a significant number of individuals would not receive ivermectin and that transmission would potentially continue challenging the expectation that onchocerciasis can be eliminated. Optimising intervention strategies in these RAPLOA 20-40% meso-endemic areas is critical as the risks are not clearly defined.

Large-scale REMO and RAPLOA surveys undertaken across Central Africa over the past decade have produced broad risk maps, and provided some insights into the geographical factors and potential challenges associated with co-endemicity. However, to fully understand the potential risks, it is important to examine data on a finer scale as prevalence can vary greatly within a short distance and according to ecological factors. For example, onchocerciasis transmission zones are considered to be up to 20kms from the riverine breeding sites of *Simulium* and its tributaries [4,14], while high loiasis transmission occurs predominately in forest and forest fringe areas. Recent studies in the Democratic Republic of Congo (DRC) have highlighted these geographical associations, and the overlapping transmission areas that potentially pose a risk [15,16].

### The Democratic Republic of Congo

The DRC is the largest endemic country for onchocerciasis and loiasis in Central Africa, and has reported SAEs associated with ivermectin for more than two decades [17]. One of the most *Loa* endemic and hence problematic areas has been the Mayombe area in the Bas-Fleuve District, Bas Congo Province. Following the launch of CDTi activities in December 2003, approximately 40 individuals reported SAEs, 28 of the cases with neurological signs showed *L. loa* encephalopathy and 19 cases developed coma with 14 deaths; 7 deaths in Kuimba, 4 in Seke-Banza and 3 in Boma rural health districts [18,19]. Consequently, the distribution of ivermectin stopped in January 2004 and an investigation was undertaken to address the high case fatality rate. The district appeared to be a high risk area for *L. Loa* area with encephalopathy related to Mf densities. The prevalence of onchocerciasis was found to be hypo to meso-endemic.

In response to these SAEs, several recommendations were made, including fine scale REMO and RAPLOA mapping, and strengthening of the infrastructure and SAE management. The mapping activity was carried out in 2004–2005, and detailed prevalence maps have recently been published [20]. The availability of these maps provides an opportunity to re-examine the onchocerciasis and loiasis prevalence distributions, identify the overlapping low and high risk areas, and determine how they geographically relate to historical co-endemic areas and the SAEs reported in the Bas Congo Province.

### Data, maps and analyses

Information related to the areas reporting SAEs in the Bas Congo Province was based on World Health Organization (WHO) reports for onchocerciasis control [18,19]. No village and individual-level data was publically available to accurately map and publish, therefore only the location of the Kuimba, Seke-Banza and Boma health districts were mapped based on the existing boundaries, and using the geographical information system ArcGIS 10 (ESRI, Redland CA). To account for potential geographical inaccuracies, a 5 km buffer was set around these health districts and added to the boundary of each one. We acknowledge that this is a limitation, however, given that the location of SAEs and death may be different to that of transmission and infection, these health districts boundaries are considered to be important in defining the high risk or geographical SAE areas within this province, and will help facilitate the response to any SAEs within the health facilities.

Data on the distribution of loiasis and onchocerciasis in the Bas Congo Province were based on the study carried out by Tekle *et al*. [20]. The prevalence of both diseases was measured simultaneously at 144 sites (villages) using the REMO and RAPLOA survey tools, and examined by three levels of endemicity; <20%, 20 to 40%, ≥40%. The published maps were imported into ArcGIS 10 and each study site/village was digitised and geo-referenced to recreate the study sites. Prevalence maps were reproduced, and the specific sites that had both low loiasis and onchocerciasis prevalence (<20%; hypoendemic), or both medium- high prevalence (≥20%; meso-hyperendemic) were highlighted and examined in relation to the historical overlapping transmission zone by Fain [21], which was recently reproduced and published [16].

All prevalence distributions were examined in relation to the Kuimba, Seke-Banza and Boma health districts and referred to as SAE areas. For each of the three prevalence levels, the number of study sites that geographically overlapped in these three SAE areas were quantified and compared to those sites that did not. Statistical differences between the prevalence levels in and out of SAE areas were first examined using the Pearson’s Chi-squared test with p-values < 0.05 considered significant. A univariate logistic regression model was then fitted to the binary SAE area data in R software to estimate the odds ratios (OR) and 95% confidence intervals (CIs) of a site being in a SAE area.

### Filarial distributions associated with SAEs

**Figure 1:**
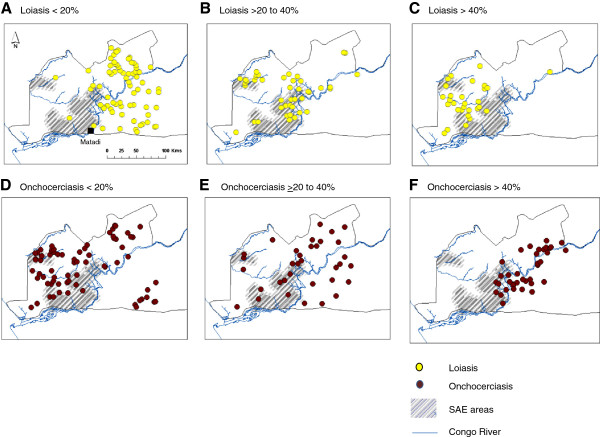
**Geographical patterns of loiasis and onchocerciasis prevalence levels in relation to health districts reporting SAEs.** Grey shading indicates SAE areas. Yellow dots indicate loiasis prevalence **A**. <20% **B**. 20 to 40% **C**. >40%. Brown dots indicate onchocerciasis prevalence **D**. <20% **E**. 20 to 40% **F**. >40%.

The location of the three health districts reporting SAEs and the different geographical patterns of the loiasis and onchocerciasis prevalence levels are shown in Figure 1. Overall, a statistically significant difference was found between the three loiasis prevalence levels and the extent to which the sites occurred in and out of the defined SAE areas (p-values <0.01), whereas no significant difference was found between the onchocerciasis prevalence levels and the SAE areas (p-value = 0.75) (Table 1).

**Table 1:** Summary of prevalence distribution by status of SAE area

	**SAE area**					
	**Yes**		**No**			
	**N**	**%**	**N**	**(%)**	**Total**	**p-value**
**Loiasis**						
0-20%	4	5%	67	95%	71	
20-40%	21	49%	22	51%	43	
> 40%	11	37%	19	63%	30	<0.01
**Onchocerciasis**						
0-20%	18	25%	55	75%	73	
20-40%	10	29%	24	71%	34	
> 40%	8	21%	29	78%	37	0.75
**Overlapping prevalences**						
Nil	22	24%	68	76%	90	
<20%	0	0%	27	100%	27	
≥20%	14	52%	13	48%	27	<0.01
**Total**	36	108	144			

Contingency table, plus p-value from a Pearson’s Chi-squared test.

#### Loiasis prevalence

The sites with low loiasis <20% (n = 71) had the least risk, and predominately occurred in the north and eastern areas of the Congo River (Figure 1A), with only 4 sites (5%) found in SAE areas (Table 1). In contrast, the greatest risk occurred in sites with medium loiasis 20 to 40%, (n = 43), which was predominantly in the central area, with half the sites in the defined SAE areas (n = 21; 49%) (Figure 1B). Compared to the low loiasis sites the odds of a site with loiasis 20 to 40% being in the SAE area was 16 times higher (OR 15.99, 95% CI 5.41-59.48) as shown in Table 2. Similarly, sites with high loiasis ≥40% (n = 30) had a greater risk with approximately one third of sites (n = 11; 37%) found in SAE areas (Figure 1C), and the odds of being in the SAE area was 10 times higher (OR 9.7, 95% CI 2.96-38.28) compared to low loiasis sites (Table 2).

**Table 2:** Regression model

	**Odds ratio**	**95% CI**	**p-value**
**Loiasis**			
0-20%	-	-	-
20-40%	15.99	(5.41, 59.48)	<0.01
40%	9.70	(2.96, 38.28)	<0.01
**Onchocerciasis**			
0-20%	-		
20-40%	1.27	(0.50, 3.13)	0.60
≥40%	0.84	(0.31, 2.12)	0.72
**Overlapping prevalences**			
Nil	-	-	-
<20%	0.00		0.99
≥20%	3.33	(1.36, 8.26)	0.01

Results obtained by fitting univariate logistic regression models to the SAE area data.

#### Onchocerciasis prevalence

The sites with low onchocerciasis prevalences <20% (n = 73) were more widely distributed, predominately found in the western area of the Congo River (Figure 1D), with 18 sites (25%) found in SAE areas (Table 1). Notably, the low prevalence onchocerciasis areas geographically overlapped those with medium to high loiasis endemicity (Figure 1B, C, D). This differed from the sites with medium onchocerciasis 20 to 40% (n = 34), which were broadly distributed in the central and eastern areas (Figures 1E) where 10 sites (29%) were found in SAE areas. In contrast, the high onchocerciasis ≥40% sites (n = 37) were found in close proximity to the Congo River with 10 sites (29%) in SAE areas, and found to predominately overlap with medium level prevalence loiasis areas (Figure 1F). Overall, the proportion of onchocerciasis sites overlapping in SAE areas was low and no statistically significant relationship was found in the logistic regression model (Table 2).

#### Overlapping high and low risk areas

**Figure 2:**
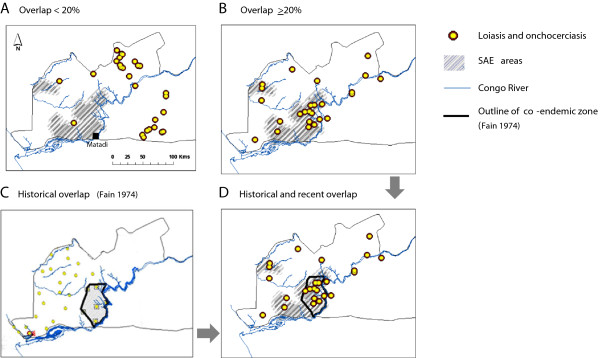
**Geographical patterns of overlapping high and low loiasis and onchocerciasis prevalence levels in relation to historical co-endemicity and health districts reporting severe adverse reactions.** Grey shading indicates SAE areas. Yellow dots with brown perimeter indicate overlapping loiasis and onchocerciasis prevalence **A**. <20% **B**. >20%. Historical prevalence and co-endemic zones are outlined in **C**, and the geographical relationship between historical co-endemic zones, overlapping prevalences of >20% and SAE areas highlighted in **D**.

Of the 144 study sites, 90 sites had a combination of low or high prevalence, with 27 sites recording low <20% prevalence, and 27 sites recording medium to high >20% prevalence of both diseases. Statistically significant differences were found between the different combinations of prevalence and the extent to which sites occurred in and out of the SAE areas (Table 1). The majority of low prevalence sites occurred in the north and eastern areas of the Congo River region with no sites found in SAE areas (Figure 2A).In contrast, sites with high ≥20% overlapping prevalence, occurred mainly in close proximity to the Congo River with 15 sites (60%) found in SAE areas (Figure 2B). Regression models suggest a significant relationship with the overlapping high prevalence sites, however, this risk cannot be fully quantified as models that combine both loiasis and onchocerciasis prevalence (both linearly, and with the inclusion of an interaction term) result in onchocerciasis prevalence being non-significant.

The comparison with historical overlapping transmission maps defined by Fain [21], (Figure 2C), showed a significant geographical overlap with the sites with high prevalence of both diseases, as well as the SAE areas (Figure 2D).

## Conclusions

These findings highlight that medium to high RAPLOA areas were most significantly associated with defined SAE areas, and estimated to be 10 to 16 times greater than low prevalence areas of *L. loa* based on the RAPLOA methodology. In particular, the sites with 20 to 40% loiasis prevalence had the greatest risk, which has major implications for APOC, as these areas are not usually considered at high risk of SAEs under ivermectin mass treatment, given that only a small proportion of the population are estimated to have high Mf *L. Loa* densities. A better understanding of the spatial distribution and relationship between RAPLOA medium/intermediate (20-40%) prevalence and high Mf densities is critical in order to maximize the distribution of safe and effective treatment in targeted onchocerciasis areas. It also raises the importance of maintaining a systematic geo-referenced database comprising village and individual level information on SAEs, so the local demographic and environmental risks within DRC can be understood with the aim of preventing or managing such catastrophic events.

The re-mapping of these co-endemic sites also provides new insights into the unique distributions of each disease. The new maps specifically highlight the contrasting spatial patterns of the high prevalence areas, and in particular the close association of onchocerciasis >40% with the Congo River. Perhaps most importantly to the new onchocerciasis ‘*elimination’* strategy is the fact that onchocerciasis hypoendemic areas geographically coincided with medium to high RAPLOA areas. This implies that ivermectin MDA cannot be extended to these drug-naive populations due to the high risk of SAEs. Therefore, different approaches such as the new *L. loa* test and treat strategy using the Cellscope to detect individuals with high Mf densities and exclude them from treatment may be safer and more economical [22]. Otherwise alternative interventions such as integrated vector management (IVM), including targeted *Simulium* and *Chrysops* spp. control, or the use of anti *Wolbachia* therapy using doxycycline [23,24] may be essential in order to achieve elimination of onchocerciasis in this particular area of DRC.

The geographical concordance between historical and recent high transmission zones (spanning three decades), suggest that these particular co-endemic areas are relatively stable and linked to distinct local characteristics. Importantly, here we show that they also geographically coincide with the defined SAE areas, which are not located in the dense forested areas where high loiasis dominates [20], nor along the Congo River where high onchocerciasis dominates - but somewhere in between - in the intermediate transmission zone where neither parasite appears to dominate. This raises important questions about parasite co-existence and competition [25], and the role of environmental and demographic factors influencing the main vectors, and the intensity and overlap of transmission.

The risk of SAEs must also be assessed in the context of the lymphatic filariasis (LF) programme in DRC, which is currently conducting baseline prevalence mapping in preparation for the scale up of MDA activities. Standard MDA includes ivermectin and albendazole [2], however, in areas where LF is co-endemic with *L. loa* the national programme will need to implement the recommended alternative strategy of twice yearly albendazole and bed nets/long-lasting insecticidal nets (LLINs) in order to reduce the risk of SAEs [26]. In selected areas further mapping at a micro-level is necessary to ensure the most appropriate interventions are distributed to those most at risk. This study provides some important insights into overlapping filarial infections and the risk of SAEs, which may help the national LF programme as it moves to treat millions of people for LF over the next few years. Coordinated and collaborative efforts between the two filarial elimination programmes are crucial to their success.

## Competing interests

The authors declare that they have no competing interests.

## Authors’ contributions

LKH and DHM conceived the idea for the paper and analysis. JC assisted with mapping. MCS carried out the statistical analysis. LKH wrote the first draft of the manuscript and all authors contributed to subsequent drafts, and agreed to the final version.
